# Effects of Different Sources of Nitrogen on Endophytic Colonization of Rice Plants by *Azospirillum* sp. B510

**DOI:** 10.1264/jsme2.ME17186

**Published:** 2018-09-29

**Authors:** Kamrun Naher, Hiroki Miwa, Shin Okazaki, Michiko Yasuda

**Affiliations:** 1 Biological Production Science, Graduate School of Agriculture, Tokyo University of Agriculture and Technology 3–5–8 Saiwai-cho, Fuchu, Tokyo 183–8509 Japan

**Keywords:** *Azospirillum* sp. B510, rice, endophytic colonization, nitrogen, acidification

## Abstract

*Azospirillum* sp. B510, a free-living nitrogen-fixing bacterium isolated from the stems of rice (*Oryza sativa* cv. Nipponbare), was investigated to establish effective conditions for the colonization of rice plants. We analyzed the effects of the nitrogen sources KNO_3_, NH_4_Cl, urea (CO[NH_2_]_2_), and NH_4_NO_3_ at different concentrations (0.01–10 mM) on this colonization. Nitrogen promoted plant growth in a concentration-dependent manner, with minor differences being observed among the different nitrogen sources. Bacterial colonization was markedly suppressed on media containing NH_4_^+^ concentrations higher than 1 mM. Since concentrations of up to and including 10 mM NH_4_^+^ did not exhibit any antibacterial activity, we analyzed several factors affecting the NH_4_^+^-dependent inhibition of endophytic colonization, including the accumulation of the reactive oxygen species H_2_O_2_ and the secretion of the chemotactic substrate malic acid. The accumulation of H_2_O_2_ was increased in rice roots grown on 1 mM NH_4_Cl. The amounts of malic acid secreted from NH_4_-grown rice plants were lower than those secreted from plants grown without nitrogen or with KNO_3_. Although the bacterium exhibited chemotactic activity, moving towards root exudates from plants grown without nitrogen and KNO_3_-grown plants, this activity was not observed with root exudates from NH_4_^+^-grown plants. NH_4_^+^, but not NO_3_^−^, caused the acidification of growth media, which inhibited plant bacterial colonization. These NH_4_^+^-dependent phenomena were markedly suppressed by the stabilization of medium pH using a buffer. These results demonstrate that the type and concentration of nitrogen fertilizer affects the colonization of rice plants by *Azospirillum* sp. B510.

Biofertilizers have been widely used in many countries as an alternative to chemical fertilizers in order to increase soil fertility and crop production for sustainable farming. The application of beneficial microbes can enhance plant growth and resistance to adverse environmental stresses, such as water and nutrient deficiencies and heavy metal contamination. One group of these microbes is referred to as plant growth-promoting rhizobacteria (PGPR), and some are commercially used as biofertilizers (6, 37).

*Azospirillum* sp., a well-studied member of PGPR, has been found in association with some of the world’s most staple food crops, including rice, maize, sorghum, wheat, and millet (14, 19, 28, 34). Members of the genus *Azospirillum* are widespread in soil and their inoculation on cereals and forage crops results in yield increases in many field experiments, not only due to nitrogen fixation, but also through the production of plant growth-promoting substances, such as the phytohormones indole-3 acetic acid (IAA) and gibberellic acid (2, 22). The inoculation of *A. brasilense* into *Zea mays* and *Sorghum biocolor* has been reported to enhance the uptake of mineral ions (NO_3_^−^, K^+^, and H_2_PO_4_^−^) (28). The uptake of NH_4_^+^ and PO_4_^−^ was also enhanced in rice plants after an inoculation with *A. lipoferum* under hydroponic conditions (31).

*Azospirillum* sp. B510 (B510) is a diazotrophic endophyte that has been isolated from the stems of a rice plant (*Oryza sativa* cv. Nipponbare) (13). Increased seed production by B510-colonized rice plants was demonstrated under greenhouse, paddy field, and laboratory conditions (7, 21, 37). Moreover, rice plants inoculated with this bacterium had induced resistance against rice blast disease and rice blight disease (42). Kaneko *et al.* (23) determined the complete genome sequence of B510; it has a single chromosome and six plasmids encoding 3,416 putative proteins, including putative genes encoding enzymes related to IAA biosynthesis and the reduction of host ethylene levels. A comparative metabolomic analysis revealed that rice plants inoculated with B510 induced a modified metabolic response in shoots and roots, suggesting that this bacterium triggers a systemic response against pathogens (7). Consequently, the beneficial effects of B510 colonization in rice make it a useful biofertilizer. However, these beneficial effects were found to vary in field experiments, depending on both the rice genotype and nitrogen level (37). Moreover, neither the mechanism by which this bacterium colonizes host plants nor the relationship between colonization and environmental conditions around the roots has been elucidated.

Nitrogen fertilizers, particularly ammonium, the form of nitrogen favored by rice, are widely used in rice cultivation; however, the influence of nitrogen fertilizers on the establishment of endophytic colonization by bacteria, such as B510, has not yet been clarified. Thus, to understand the relationship between nitrogen nutrition and endophytic colonization by B510 in rice plants, we performed physiological, histochemical, and microscopic analyses on rice-*Azospirillum* interactions. We found that high concentrations of NH_4_^+^ exert indirect suppressive effects on the colonization of rice plants by B510. We also propose a model to explain the mechanisms responsible for the effects of high NH_4_^+^ concentrations on the plant redox state, rhizosphere acidification, bacterial chemotaxis, and host colonization by B510.

## Materials and Methods

### Bacterial strains and growth conditions

The bacterial strains used in the present study are listed in Table S1. *Azospirillum* sp. B510 was grown at 28°C in nutrient broth (NB) medium (Eiken Chemical, Tokyo, Japan) with appropriate antibiotics (50 μg mL^−1^ polymyxin and 50 μg mL^−1^ streptomycin). *Escherichia coli* strain S17-1 was grown at 37°C in Luria-Bertani (LB) medium supplemented with 50 μg mL^−1^ ampicillin. DsRed-labeled *Azospirillum* sp. B510 was constructed using the plasmid pBjGroEL4::DsRed2, as described by Hayashi *et al.* (18).

### Plant growth conditions and endophytic inoculation

Seed coats were removed from rice seeds (*O. sativa* cv. Nipponbare), which were then surface-sterilized with 70% ethanol for 30 s and shaken in 5% (w/v) sodium hypochlorite (Wako Pure Chemical Industries, Osaka, Japan) for 10 min. Seeds were then washed with sterilized distilled water three times for 10 min each time.

Regarding seed inoculation, B510 was grown on NB medium including 50 mg L^−1^ polymyxin B (Wako Pure Chemical Industries) at 28°C, harvested at 30 h, and washed twice with sterilized distilled water. Bacterial cells were resuspended in sterilized distilled water to a final density of 10^9^ colony-forming units (CFU) mL^−1^ (OD_600_=1.0). Bacterial suspensions were diluted 100-fold in sterilized distilled water to a final density of 2×10^7^ CFU mL^−1^.

Sterilized seeds were transferred into plant boxes containing 100 mL of semisolid rice growth (RG) medium adjusted to pH 5.5 (13) with various concentrations of nitrogen and were immediately inoculated with B510 (50 μL seed^−1^). Potassium nitrate (KNO_3_), ammonium chloride (NH_4_Cl), urea (CO[NH_2_]_2_), and ammonium nitrate (NH_4_NO_3_) were obtained from Wako Pure Chemical Industries and the absence of a nitrogen source acted as a control. Stock solutions (prepared in distilled water at a concentration of 1 M) were then filtered through a 0.22-μm membrane filter (Millex-GP; Merck Millipore, Damstadt, Germany). Plant boxes (CUL-JAR300; Iwaki, Tokyo, Japan) containing the inoculated seeds were incubated in a plant growth chamber (LPH-240SP; NK System, Osaka, Japan) under 16-h light:8-h dark conditions at 25°C for 7–10 d depending on the individual experiment.

### Estimation of the population of *Azospirillum* sp. B510 inside rice tissues

To estimate the population of B510 inside rice tissues, whole 10-d-old seedlings of *O. sativa* cv. Nipponbare previously inoculated with the bacteria were weighed and then surface-sterilized with 70% ethanol for 15 s and subsequently with 1% sodium hypochlorite (NaOCl) for 30 s. The seedlings were then quickly washed five times with sterilized distilled water. The seedlings were homogenized in 0.8% NaCl using a sterilized mortar and pestle and the homogenate was plated on NB agar containing 50 mg L^−1^ polymyxin B at appropriate dilutions. After an incubation at 28°C for 3 d, the number of polymyxin B-resistant bacterial colonies was counted.

### Microscopy

Regarding fluorescence microscopy, rice seedlings inoculated with DsRed–labeled *Azospirillum* sp. B510 were cultivated for 10 d, sampled from the agar plant box, and washed gently with sterilized distilled water. The roots were placed separately on microscope slides. They were observed and photographed using an Olympus IX71 fluorescence stereomicroscope (Olympus, Tokyo, Japan). Each field was observed under three different conditions: optical light microscopy, fluorescence microscopy with a GFP field, and fluorescence microscopy with a Ds-Red field to confirm red fluorescence from DsRed–labeled *Azospirillum* sp. B510.

### DAB staining

3,3′-Diaminobenzidine (DAB) staining was performed according to the procedures described by Fester and Hause (15) with the following modifications. To visualize hydrogen peroxide (H_2_O_2_), rice plants were placed in DAB solution (1 mg mL^−1^ DAB; Tokyo Chemical Industry, Tokyo, Japan) buffered with 200 mM sodium phosphate, pH 6.5, at room temperature for 3–4 h. Photographs were taken using a Lumix DMC-G3 camera (Panasonic, Osaka, Japan). Quantitative measurements of H_2_O_2_ were analyzed as described previously (43). Briefly, DAB-stained root samples (100 mg) were washed in 80% ethanol for 20 min and homogenized immediately in 500 μL of 0.2 M perchloric acid (HClO_4_) (Wako Pure Chemical Industries) in a pre-cooled mortar. The mixtures were incubated on ice for 5 min and then centrifuged (10,000×*g*, 4°C, 10 min). The absorbance of the supernatants was measured at 450 nm and H_2_O_2_ concentrations were obtained via a standard calibration with solutions of 0.2 M HClO_4_ containing 5, 10, 25, and 50 μM H_2_O_2_ (Sigma-Aldrich, St. Louis, MO, USA).

### Measurement of malic acid in root exudates

Rice plants were grown in RG medium inoculated with or without B510 for 10 d. Each seedling was washed gently with sterilized distilled water and placed into 30 mL Milli-Q water in a 50-mL Falcon^TM^ tube for 2 d in the growth chamber. After the removal of seedlings, the solution was filtered with a 0.22-μm membrane filter (Millex-GP; Merck Millipore), lyophilized, and stored at −80°C. The lyophilized sample was dissolved in 1 mL Milli-Q water before being analyzed.

A malic acid analysis was performed using the series LC-20AC HPLC system (Shimadzu, Kyoto, Japan) with a Shodex RSPAK KC-811 (300×8 mm) analytical column and KC-811 pre-column (Showa Denko K.K., Tokyo, Japan) run with 0.1% phosphate buffer at a flow rate of 0.25 mL min^−1^. The column temperature was set to 40°C. Malic acid was detected and quantified using a UV detector set at 210 nm. The peaks obtained were compared with an array of standard malic acid peaks run under the same conditions. The major peak was identified by comparing the retention time with that of the matching standard. Standard malic acid (Sigma-Aldrich) and root exudates (25 μL of each) were sequentially injected into the chromatographic system and run under the same conditions with four replicates per sample. Malic acid in the root exudate was identified by comparisons with the retention times of standard samples.

### Chemotaxis assay

The drop assay was performed as described in de Weert *et al.* (10) with slight modifications. B510 was grown in NB medium at 28°C and 160 rpm until the logarithmic phase (OD_600_ of 0.8) was obtained. Bacterial cells were washed and resuspended in sterilized distilled water to an OD_600_ of 2.0 and 1% hydroxypropylmethylcellulose solution (Sigma-Aldrich) was then added (final concentration, 0.25%). The cell suspension was transferred to a 90-mm Petri dish, on which it formed a 3-mm-thick layer. Concentrated (50-fold) root exudates or individual 100 mM organic acid components were added to the center of the dish as a 10-μL drop. After an incubation at room temperature for 15 min, the plates were inspected for the appearance of a clear zone surrounding the drop.

### Biofilm assay

The production of biofilms by *Azospirillum* sp. B510 was measured *in vitro* using a PVC microtiter plate assay (40). Five μl of B510 cultures adjusted to OD_600_=0.01 were inoculated into 95 μL of NB medium a 96-well microtiter plate (TPP Techno Plastic Products AG, Trasadingen, Switzerland), which was then incubated without shaking at 28°C for 48 h. In the quantification of biofilm development, 25 μL of 1.0% crystal violet solution (Wako Pure Chemical Industries) was added to the wells. After a 30-min incubation, unbound crystal violet in each well was gently removed with a pipette and the wells were washed with distilled water, followed by 70% ethanol, and then distilled water. Crystal violet in each well was solubilized by adding 100 μL of 100% ethanol and quantified by absorbance at 550 nm.

### pH measurement

The pH of the cultivation medium was adjusted to 5.5 before cultivation. After cultivation for 7 d, RG medium was removed from the plants and mixed thoroughly. The pH of the medium was measured using a pH meter (Horiba, Kyoto, Japan). RG medium containing 0.05% bromophenol blue (BPB, Wako Pure Chemical Industries) was used to assess pH. To maintain pH in RG medium at 5.5, 100 mM of 2-morpholinoethanesulfonic acid monohydrate (MES) (Wako Pure Chemical Industries) was added to RG medium containing NH_4_Cl.

### Statistical analysis

Statistical analyses were performed using a one-way analysis of variance (ANOVA) followed by the Student-Newman-Keuls (SNK) test. Different lower-case letters represent significantly different values (*P*<0.05).

## Results

### Effects of various nitrogen sources on endophytic colonization of rice by *Azospirillum* sp. B510

**Fig. 1:**
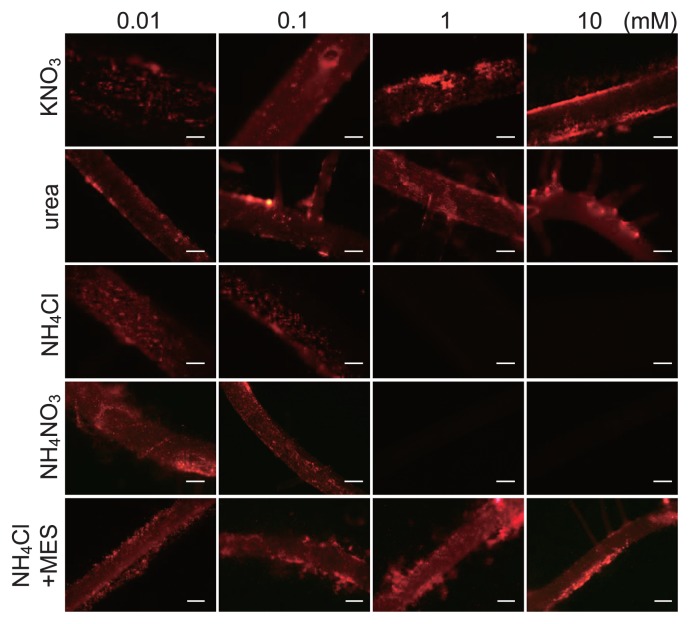
Epiphytic colonization of rice roots by DsRed-labeled *Azospirillum* sp. B510. Colonization by B510 was observed using fluorescence microscopy (Olympus, BX50). Micrographs of 10-d-old lateral roots grown on RG medium containing different sources and concentrations of nitrogen are as indicated. Scale bars=100 μm.

Seedlings of *O. sativa* cv. Nipponbare were inoculated with the DsRed-labeled B510 strain to establish optimum conditions for endophytic colonization. Plants were grown in RG medium containing different concentrations (0.01–10 mM) of each nitrogen source (KNO_3_, urea, NH_4_Cl, or NH_4_NO_3_). In most cases, rice roots inoculated with DsRed-labeled B510 showed strong red fluorescence under the fluorescence microscope (Fig. 1). Marked differences were not observed between 0.01 and 0.1 mM of each nitrogen source. Rice plants grown in more than 1 mM KNO_3_ or urea exhibited strong fluorescence, indicating well-established bacterial colonization on root surfaces (epiphytic colonization). In contrast, red fluorescence was not detected in plants grown at NH_4_Cl and NH_4_NO_3_ concentrations of 1 mM or higher, suggesting that NH_4_^+^ suppressed bacterial colonization (Fig. 1). To further confirm these results, we counted the number of B510 cells on root surfaces. The number of B510 cells on root surfaces was 100-fold lower in 1 mM NH_4_Cl-treated plants and 10,000-fold lower in 10 mM NH_4_Cl-treated plants than in control plants without NH_4_Cl (Fig. S1).

**Fig. 2:**
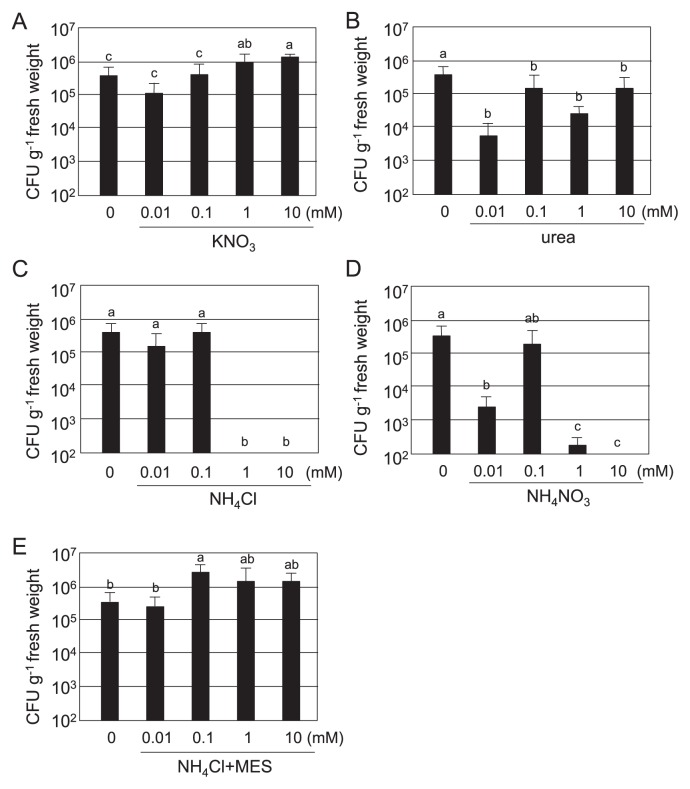
Endophytic colonization of rice plants grown with different nitrogen sources by *Azospirillum* sp. B510. (A) KNO_3_, (B) urea, (C) NH_4_Cl, (D) NH_4_NO_3_, (E) NH_4_Cl+100 mM MES buffer. Seeds were treated with B510 (final concentration, 2×10^5^ CFU plant^−1^). Whole plants were surface sterilized and homogenized 10 d after the inoculation. The number of CFU was estimated by their growth on nutrient broth agar plates containing 50 μM polymyxin. Values presented are the average±SD from four replicates of one plant each. Different letters indicate significant differences between treatments (Student-Newman-Keuls [SNK] test, *P*<0.05, *n*=4).

We then examined the endophytic colonization of rice by B510 by counting the number of colonies within surface-sterilized whole plants 10 d post-inoculation (Fig. 2). In rice plants grown on KNO_3_, the number of B510 cells became higher as the KNO_3_ concentration increased (Fig. 2A). Although the root length was very short in RG medium containing 10 mM urea, endophytic colonization was clearly detected (Fig. 2B and S2). B510 colonized the inside of host roots with 10^5^–10^6^ CFU g^−1^ fresh weight at low concentrations (0.01–0.1 mM) of NH_4_Cl. However, endophytic colonization by B510 was markedly suppressed at high concentrations (higher than 1 mM) of NH_4_Cl with no detection (Fig. 2C). B510 also colonized host roots with 10^3^–10^5^ CFU g^−1^ fresh weight at a low NH_4_NO_3_ concentration (0.01–0.1 mM), and only 10^2^ CFU g^−1^ fresh weight or no detection at 1 or 10 mM NH_4_NO_3_, respectively. These results indicate that high concentrations of both forms of ammonium (NH_4_Cl and NH_4_NO_3_) have a negative effect on endophytic colonization by B510.

Since a high concentration of NH_4_^+^ is known to have a toxic effect on bacteria (3, 29), we investigated whether NH_4_^+^ inhibits the growth of B510 at the concentrations used in the colonization experiments. B510 was incubated in RG medium adjusted to pH 5.5 containing each concentration (0.01, 0.1, 1, and 10 mM) of NH_4_Cl. No significant differences were observed in bacterial growth among these concentrations of NH_4_Cl, demonstrating that NH_4_^+^ did not exhibit any antimicrobial activity against B510, which is in contrast to findings obtained using other bacteria (Table S3). These results indicate that endophytic colonization by B510 was inhibited by high concentrations of NH_4_^+^, but also that high concentrations of NH_4_^+^ did not inhibit B510 growth *in vitro*. Bacterial exopolysaccharides (EPS) are important for the attachment of the endophyte to the root surface (30). The establishment of a biofilm structure involves bacterial cells and EPS, which produce an optimal biosphere for the conversation of genetic material between cells. Thus, biofilms are of great importance in the plant-microbe interaction. The quantity of EPS in biofilms may comprise approximately 50–90% of organic compounds (11). However, B510 produced EPS or biofilms at similar levels regardless of the nitrogen source (NH_4_+ or NO_3_; Fig. S4A and B). This result suggests that EPS or biofilm-mediated attachment is not a key step in the NH_4_^+^-dependent suppression of colonization.

### NH_4_Cl induces H_2_O_2_ accumulation in rice roots

**Fig. 3:**
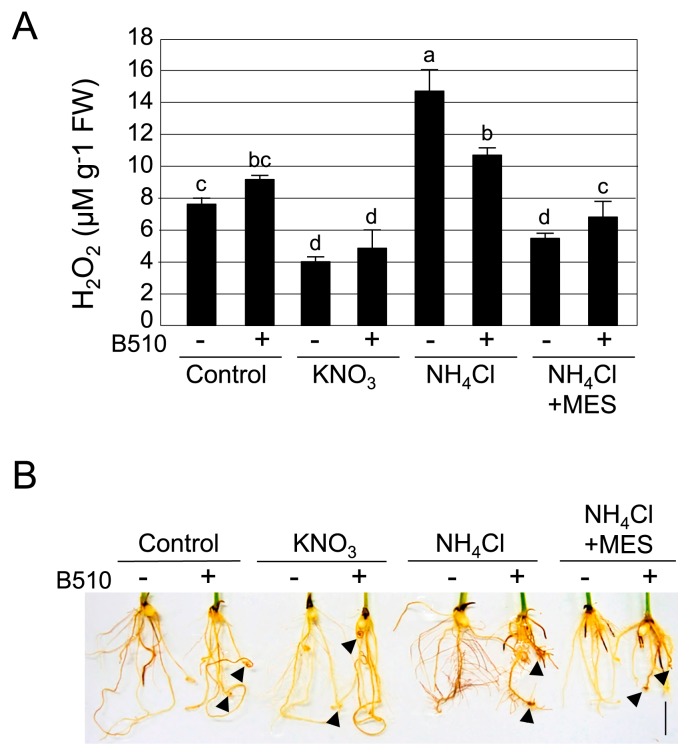
Qualitative and quantitative assessments of H_2_O_2_ in B510-inoculated rice grown on RG medium. DAB staining was performed 10 d after the inoculation of rice seeds with B510. (A) The accumulation of H_2_O_2_ was quantified in the roots using standard H_2_O_2_ concentrations to calibrate data during optical density (OD) measurements. Plants were grown in RG medium including 1 mM of KNO_3_, 1 mM NH_4_Cl, 1 mM NH_4_Cl+100 mM MES, or without nitrogen (control), inoculated with B510 (+) or not inoculated (−). Error bars indicate standard deviations (*n*=3). (B) H_2_O_2_ levels correlate with color intensity (brown). The arrow indicates root curling. Scale bar=0.5 cm.

Plant-associated bacteria need to cope with host defense responses during infection. Plant defense responses to pathogen infection involve the production of reactive oxygen species (ROS), including H_2_O_2_. H_2_O_2_ directly inhibits the growth of bacterial and fungal pathogens (41). Since infection by B510 was suppressed at 1 mM NH_4_^+^, we measured the accumulation of H_2_O_2_ in the roots of infected rice plants grown without nitrogen (control) and with 1 mM KNO_3_ and 1 mM NH_4_Cl using the DAB staining method (Fig. 3). The brown color that formed with DAB, which correlates with H_2_O_2_ accumulation, was more intense in roots that were grown in NH_4_Cl than in KNO_3_ and the control (Fig. 3B). Quantitative measurements of H_2_O_2_ revealed that NH_4_Cl induced significantly greater H_2_O_2_ accumulation than KNO_3_ (Fig. 3A). These results suggest that H_2_O_2_ accumulated with the NH_4_Cl treatment, but that its production was slightly reduced by the inoculation with B510.

### Chemotactic responses to root exudates from plants grown with KNO_3_ or NH_4_Cl

**Fig. 4:**
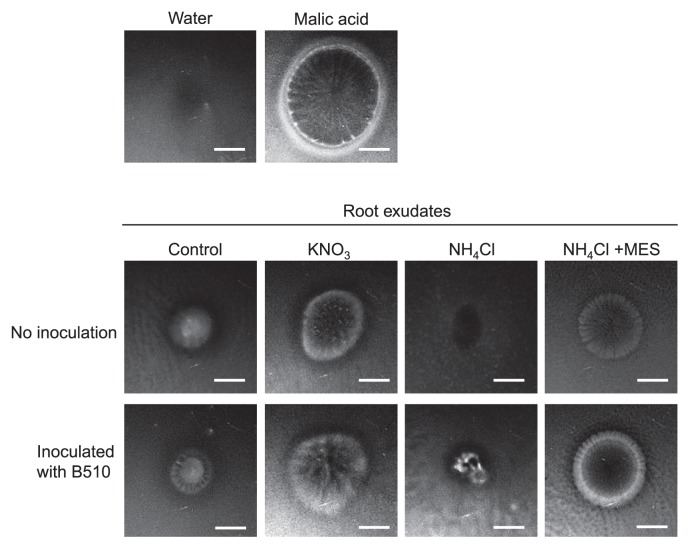
Chemotactic response of B510 towards rice root exudates. The chemotactic response of B510 was analyzed using a negative control (water), positive control (100 mM Malic acid), and root exudates from rice roots. Rice seedlings were grown in RG media including control, 1 mM KNO_3_, 1 mM NH_4_Cl, or 1 mM NH_4_Cl+100 mM MES for 10 d without an inoculation or an inoculation with B510. Before transplantation, seedlings were washed twice in sterilized distilled water and transplanted to 30 mL sterilized distilled water for 2 d. A 50-fold concentrated root exudate (10 μL) was added to the center of each Petri dish (90 mm). The bacterial chemotactic response was triggered after an incubation at room temperature for 10 min. A response correlated with the appearance of a ring of turbidity near the center of each Petri dish. Scale bars=1 cm. The experiment was repeated three times with similar results.

*Azospirillum* strains display chemotactic responses to specific substrates, such as root exudates, including organic acids (33). To analyze chemotactic responses to root exudates secreted from rice roots grown in RG media without nitrogen (control) and with KNO_3_ and NH_4_Cl, we performed a taxis assay (Fig. 4). Root exudates extracted from control and KNO_3_-grown roots resulted in the formation of a bacterial ring in the plate assay, revealing a positive chemotactic reaction. However, the bacterial ring was not observed when root exudates from NH_4_Cl-grown roots were used (Fig. 4). The bacterial rings formed by the root exudates of B510-inoculated plants were slightly larger than those of the non-inoculated control, suggesting that the B510 inoculation stimulated chemoattractant production by roots (Fig. 4, lower panels).

### Measurement of malic acid in root exudates from plants grown with KNO_3_ or NH_4_Cl

**Fig. 5:**
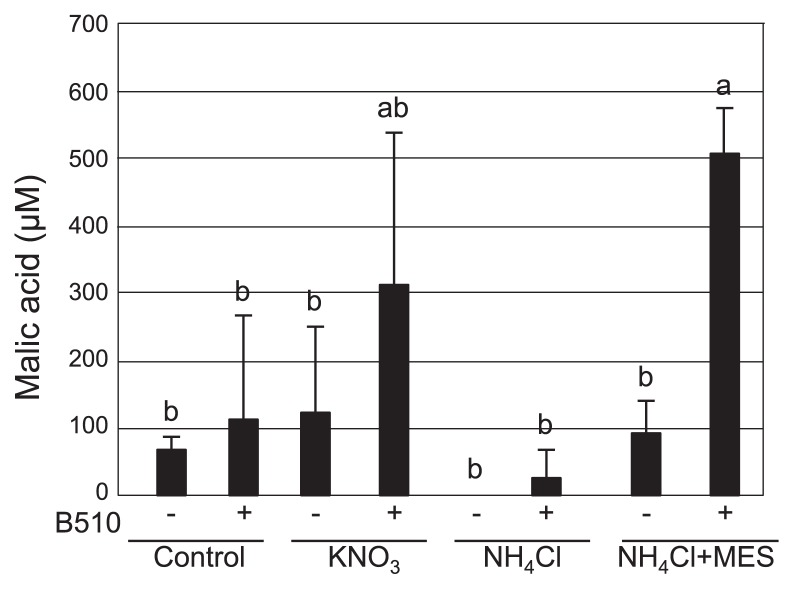
Measurement of malic acid in root exudates. Root exudates were extracted from 10-d-old seedlings grown in RG medium including 1 mM of KNO_3_, 1 mM NH_4_Cl, 1 mM NH_4_Cl+100 mM MES, or without nitrogen (control) without an inoculation (−) or inoculated (+) with B510. Malic acid concentrations were quantified by HPLC. The experiment was repeated three times with similar results.

Previous studies revealed that root-secreted malic acid (a chemotaxis substrate) recruits beneficial soil bacteria to promote binding and biofilm formation in *Arabidopsis* (35). B510 also responds to malic acid and other organic acids (Fig. S3). To investigate whether the amount of organic acids secreted by the roots increased 10 d after the B510 inoculation, we quantified malic acid secreted from the roots using HPLC. No significant differences were observed in the concentration of malic acid between control plants without the inoculation and B510-inoculated plants (Fig. 5). Although the amount of malic acid secreted from plants grown in KNO_3_ without the inoculation was similar to that of plants grown in the absence of a nitrogen source with and without the inoculation, the amount of malic acid markedly increased when the plants had also been inoculated with B510 (Fig. 5). Malic acid was not detected in the root exudates secreted from non-inoculated NH_4_Cl-grown plants, whereas a small amount was detected in the root exudates of B510-inoculated plants grown with NH_4_Cl (Fig. 5). These results indicate that the B510 inoculation slightly increased the secretion of malic acid from rice roots although no significant difference was observed between inoculated and non-inoculated plants.

### Acidification of the rhizosphere by NH_4_^+^ affects endophytic colonization by B510

**Fig. 6:**
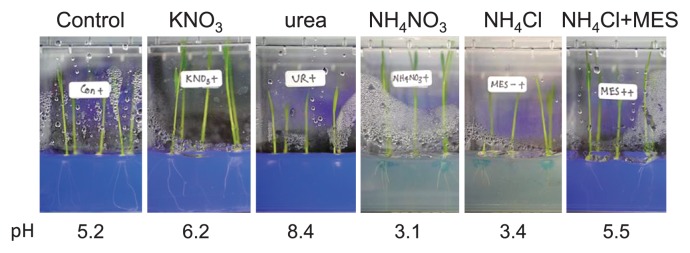
Visualization of changes in pH in RG medium containing various nitrogen sources. Rice seedlings were grown in RG medium containing bromophenol blue (BPB) including 1 mM of KNO_3_, 1 mM NH_4_Cl, 1 mM NH_4_Cl+100 mM MES, or without nitrogen (control) for 10 d. The decolorization of BPB correlated with a decrease in pH levels in RG medium. Photos were taken 7 d after planting.

Nitrogen plays a prominent role in the cation-anion balance. It may be taken up by plants as a cation (NH_4_^+^) or anion (NO_3_^−^). Previous studies demonstrated that plants supplied with NO_3_^−^ counterbalance the corresponding excess of negative charges by releasing equivalent amounts of OH^−^ or HCO_3_^−^ into the rhizosphere, thereby increasing rhizosphere pH (9, 27). Plants receiving NH_4_^+^ counterbalance the corresponding excess of positive charges by releasing equivalent amounts of H^+^ into the rhizosphere, thereby decreasing rhizosphere pH (20). Thus, we examined whether a pH change in the rhizosphere using different nitrogen sources affects the B510-endophytic colonization of rice plants. Before planting the seeds, the pH of RG medium was adjusted to 5.5. The acidification of RG medium after plant growth was clearly visualized using BPB, which changes from purple-blue to colorless when pH is between 3.0–4.6 (Fig. 6). The pH of control medium (without nitrogen) decreased slightly to pH 5.2 7 d after planting (Fig. 6). In the cases of KNO_3_ and urea, pH increased to 6.2 and 8.4, respectively. However, in the cases of NH_4_NO_3_ and NH_4_Cl (in which H^+^ is released), pH markedly decreased to pH 3 and RG medium de-colorized (Fig. 6). The inoculation with B510 did not significantly affect pH (Table S2). These results indicate that NH_4_^+^ induced acidification around the rice rhizosphere. Therefore, the low pH caused by NH_4_^+^ may inhibit endophytic colonization by B510.

To analyze the effects of NH_4_^+^-induced acidification on endophytic colonization by B510, we tested the addition of MES buffer to RG medium, which was expected to prevent a decrease in pH. Without MES buffer, the pH of RG medium containing NH_4_Cl decreased to 3.4 (Fig. 6). In RG medium containing NH_4_Cl and MES, pH was maintained at 5.5 (Fig. 6) and endophytic and root surface colonization by B510 was not inhibited under higher concentrations of NH_4_^+^ tested (Fig. 1 and 2E). Furthermore, the accumulation of H_2_O_2_ decreased in rice roots grown with NH_4_Cl and MES (Fig. 3). A chemotactic response by B510 was observed with exudates extracted from roots grown with NH_4_Cl and MES (Fig. 4). In addition, malic acid secretion was increased by the B510 inoculation in exudates from roots grown with NH_4_Cl and MES (Fig. 5). These results indicate that the pH decrease induced by NH_4_^+^ inhibited endophytic colonization by B510. We then performed a bacterial growth test using NB media adjusted to different pH levels (pH 3–8). The results obtained indicated that acidic media with pH<4 suppressed the growth of B510 (Table S4). These results imply that acidification directly affects the bacterial growth of as well as endophytic colonization by B510.

## Discussion

Endophytic bacteria invade internal plant tissues through sites of injury in the epidermis, root tips, and root cracks formed at the sites of lateral roots, and some endophytic bacteria spread to distant plant organs (5, 36). *Azospirillum* sp. B510 was isolated from the stems of rice (13). A previous study demonstrated that B510 colonized the interior of rice roots using orthogonal optical sections (7). In the present study, we observed strong red fluorescence around lateral root emergence sites by fluorescence microscopy using DsRed-labeled B510, suggesting that *Azospirillum* sp. B510 invades through this site and then spreads to stems and leaves (Fig. 1). We did not detect any surface colonization by B510 in the roots under high NH_4_^+^ concentrations (Fig. 1). The fluorescence of DsRed is known to decrease under mildly acidic conditions (pH 4.0–4.8) (39). In our experiments, high concentrations of NH_4_^+^ acidified the rhizosphere to a pH of 3.0–3.9, suggesting the quenching of DsRed under acidic conditions. Consequently, we confirmed decreased colonization by B510 on root surfaces from plants grown with NH_4_^+^concentrations higher than 1 mM using a plate dilution method (Fig. S1).

A microscopic analysis also showed the non-uniformity of colonization by B510 on the root surface (Fig. 1). A previous study on non-uniform colonization by endophytic bacteria used the term ‘carpet-like structure’ to describe its appearance (44). This appearance may be explained by numerous factors, such as various root exudation patterns and bacterial quorum sensing effects, as well as chemotactic responses, twitching motility, exopolysaccharide production, and ROS-scavenging enzymes (1, 4, 8, 29). Moreover, *Azospirillum* spp. exhibit strain-specific chemotactic responses to organic acids (33). In B510, we observed a chemotactic response to rice root exudates and organic acids, and plants inoculated with B510 secreted more chemotactic compounds than non-inoculated plants (Fig. 4, 5). This strain strongly responds to oxalic acid (Fig. S3h). The different chemotactic responses among strains has been linked to the adaptation of the bacteria to the nutrient conditions provided by the host plants (33).

Biofilms or EPS have great importance in plant microbe interaction. Biofilm integration of bacterial cells convert or adapt their habitat by generating extracellular polymeric substance (EPS) matrix, reduced growth rates, regulate the quorum sensing and the up- and down regulation of specific genes. B510 produced EPS at similar levels regardless of the nitrogen source (NH_4_^+^ or NO_3_; Fig. S4). This suggests that EPS-mediated attachment may not be a key step in the NH_4_^+^ dependent suppression of colonization. Our results suggest that B510 in the soil moves towards the host plant, and after stable colonization using EPS, induces host plants to increase their secretion of compounds such as organic acids, which the bacteria then use for their growth.

Rice plants need large amounts of nitrogen, especially for growth of above-ground tissues, such as leaves. Generally, chemical fertilizers such as NO_3_^−^, NH_4_^+^, or urea are applied directly to the plant roots in order to maximize absorption. Rice plants have the capacity to utilize both NO_3_^−^ and NH_4_^+^ (24, 26). Although the growth of above-ground rice tissues is similar with the application of either NO_3_^−^ or NH_4_^+^, in our study, the growth of roots is suppressed dramatically by high concentrations of NH_4_^+^ (Fig. S2). As shown in Fig. S2, plant growth suppression by B510-inoculation was observed under some conditions. Previous studies reported that B510 enhanced rice growth and yield (21), however, the positive effects of B510 were dependent on the plant growth condition. Sasaki *et al.* (2010) showed the negative effects of B510 on rice cv. Nipponbare underin a low nitrogen fertilizer-applied field (37). Since RG medium does not contain many nutrients, the B510 inoculation may induce stress in rice plants by competing for nutrients. Moreover, the B510 inoculation caused root tip curling (shown in Fig. 3). This morphological change may also cause the growth suppression of rice plants.

B510 did not grow under acidic conditions (pH<4), suggesting that rhizosphere acidification by NH_4_^+^ contributes to the inhibition of bacterial colonization. Plants maintain a neutral intracellular pH (pH 6–7) even when the rhizosphere is acidic (25, 32). However, in the present study, endophytic colonization by B510 was not observed when the rhizosphere was acidic. This result suggests that B510 resides in the intercellular spaces of host roots rather than intracellularly and, thus, bacterial infection is affected by extracellular pH changes. The optimal pH for the growth of B510 is pH 5–7, as is that of other *Azospirillum* sp. strains (Table S4; [12]).

Host-derived ROS are key factors controlling the bacterial infection of host plants. Endophytic bacteria possess ROS-scavenging enzymes to allow successful infection. Genome data shows that B510 has two superoxide dismutases (*sod1*: AZL_024870, *sod2*: AZL_014560) and a glutathione reductase (*NADPH*: AZL_a04110). These two enzymes are essential for endophytic colonization by *Gluconacetobacter diazotrophicus* PAL5 (1). In the present study, rice plants constitutively produced the ROS, H_2_O_2_, at higher levels under high NH_4_^+^ concentrations (Fig. 3). This result implies that B510 overcomes transient and low-level ROS production by the host plant using these types of ROS-scavenging enzymes, but also that it cannot overcome constitutive and high-level ROS production by the host under high NH_4_^+^ concentrations. However, the accumulation of H_2_O_2_ was significantly decreased in rice roots grown with NH_4_Cl and MES (Fig. 3), and endophytic colonization by B510 was not suppressed in these rice roots. These results suggest that high NH_4_^+^ concentrations inhibit colonization by B510 not only through acidification, but also with the accumulation of high levels of ROS production.

**Fig. 7:**
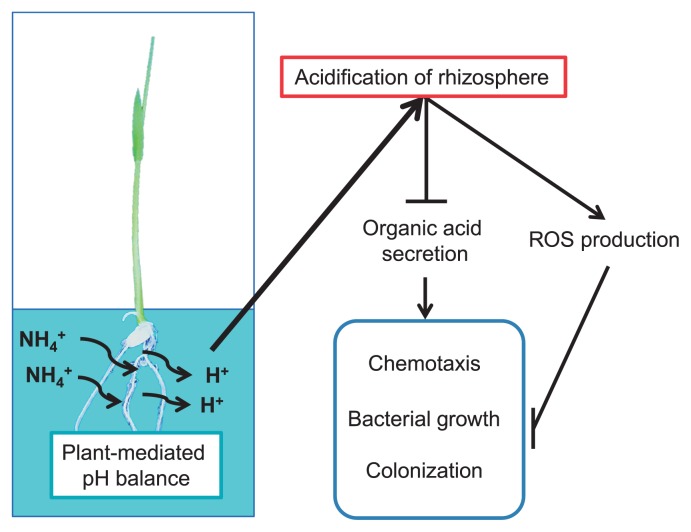
Proposed model for the suppression of bacterial colonization of rice roots by acidification with NH_4_^+^ Following the application of NH_4_^+^, rice plants induce a H^+^ efflux to maintain the pH inside their cells. The consequent acidification of the rhizosphere suppresses the secretion of chemotactic substrates, including malic acid, by the roots. As a result, the growth of bacteria, such as B510, is markedly suppressed. Moreover, acidification induces the production of ROS, including H_2_O_2_, by the plant roots. This suppresses colonization by endophytic bacteria, such as B510.

In the present study, we demonstrated that high concentrations of NH_4_^+^ inhibited the endophytic colonization of rice by B510. We propose a model to explain the mechanisms by which this phenomenon occurs (Fig. 7). In this model, root-mediated pH changes, caused by the application of NH_4_^+^, result in acidification around the rice rhizosphere. Low pH (<5.0) decreases root growth, increases H_2_O_2_ accumulation (45), and inhibits the secretion of chemotactic organic acids in root exudates, thereby suppressing the growth of and colonization by this bacterium. H_2_O_2_ production is reduced in plants grown in the presence of NH_4_^+^ and inoculated with B510 (Fig. 3). The biosynthesis of malic acid was increased in plants grown in the presence of NO_3_^−^ and inoculated with B510 (Fig. 5), and their root exudates exhibited enhanced chemotactic activity (Fig. 4). This result indicates additional benefits to those identified in previous studies, in which B510 enhanced plant growth and plant disease resistance (21, 42). These beneficial traits make B510 a superb source of biofertilizer.

Recent studies reported that rice plants fertilized with nitrogen contribute to environmental pollution (such as eutrophication) by emitting ammonia in Japanese rice paddies, which is caused by NH_4_^+^ (16, 17). In the present study, endophytic B510 cells were maintained at high numbers by controlling the pH of the rice rhizosphere using a buffer, and the roots also secreted more chemotactic compounds. Future studies will be directed towards the identification of the optimal combination of nitrogen fertilizers and B510 inoculation to promote the sustainable production of crops such as rice.

## Supplementary Material
