# Supplementary material for: Effects of Different Sources of Nitrogen on Endophytic Colonization of Rice Plants by Azospirillum sp. B510
Source: Microbes Environ. 2018 Sep 29;33(3):301–8. doi: 10.1264/jsme2.ME17186 (PMC6167112; doi:10.1264/jsme2.ME17186)
Supplement: Supplementary file 1 [file 33_301_s1.pdf]

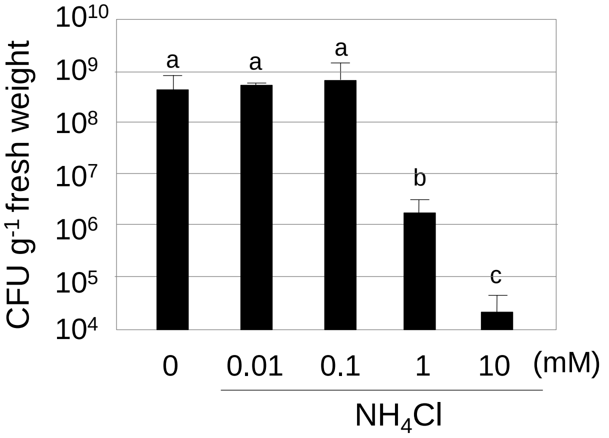

Figure S1. The colonization of rice plants by *Azospirillum* sp. B510 grown with different concentrations of NH_4Cl.

After inoculation with B510 ($2x10^5$ cfu/seed), seedlings were grown on RG media for 10 d containing different concentrations of NH_4Cl, as shown. Seedlings were transferred to 10 ml sterilized distilled water, vortexed for 30 s and the number of CFUs was estimated by growth on nutrient broth agar plates containing 50 µM polymyxin. Values presented are the average ± SD from four replicates of one plant each. Different letters indicate statistically significant differences between treatments (Student-Newman-Kuels (SNK) test, $p < 0.05$). Similar results were obtained in two independent experiments.

(Naher *et al*., 2018)

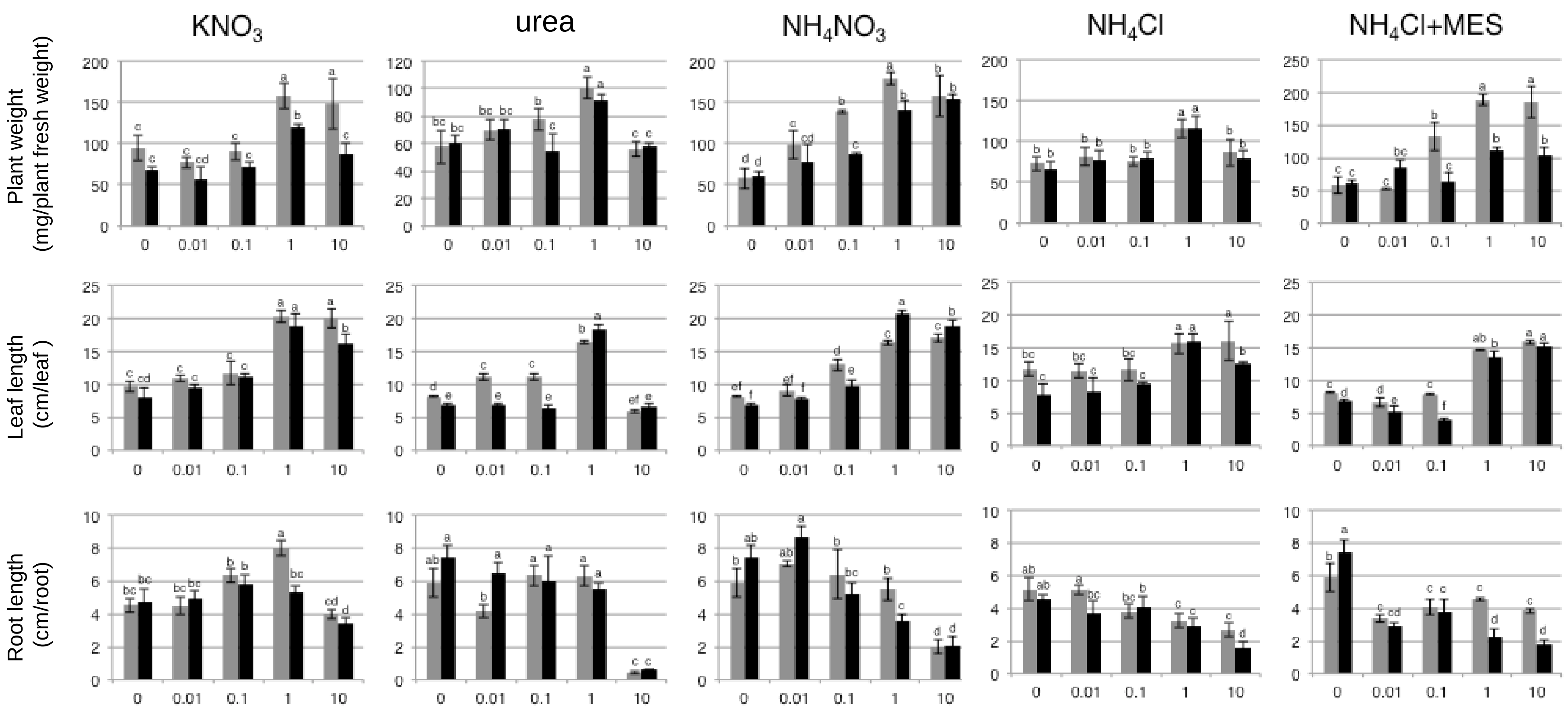

Figure S2. The effect of nitrogen sources and inoculation with B510 on rice growth.
Rice seedlings were grown in RG media containing four nitrogen sources (KNO_3, urea, NH_4Cl and NH_4NO_3) at various concentrations (0.01, 0.1, 1, and 10 mM) for 10 d. Non-inoculated controls are shown in light gray and B510-inoculated seedlings are shown in black. After washing with distilled water and removal of the soft agar, seedlings were weighed, and the weights and lengths of the leaves and roots were also measured.

(Naher *et al.*, 2018)

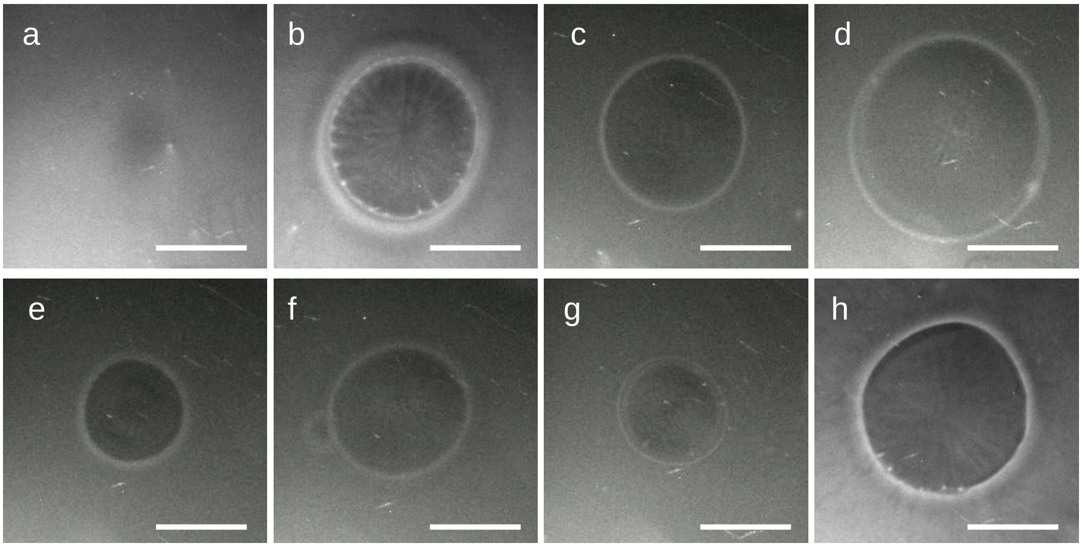

Figure S3. Chemotactic response of B510 towards organic acids.
10 µl of the organic acids, previously detected in root exudates from rice (27), were added to the center of each petri dish (90 mm diameter) which had been previously coated with bacteria (as described in Materials and Methods). The bacterial chemotactic response was triggered after incubation for 10 min at RT. The response correlated with the appearance of a ring of turbidity near the center of each Petri dish. (a) water (control), (b) 100 mM malic acid, (c)100 mM maleic acid, (d) 100 mM citric acid, (e) 100 mM acetic acid, (f) 100 mM succinic acid, (g) 100 mM lactic acid, and (h) 100 mM oxalic acid. Scale bar = 1 cm. The experiment was repeated three times with similar results.

(Naher *et al*., 2018)

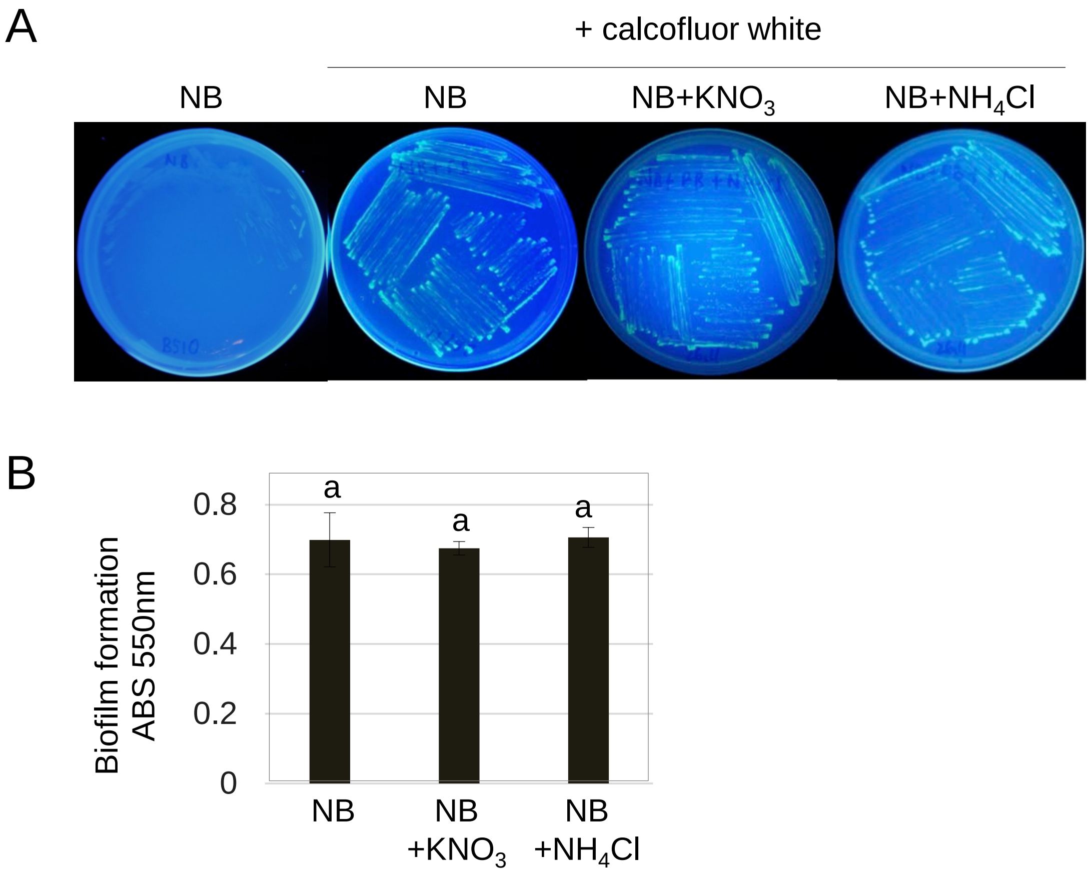

Figure S4. EPS and biofilm production by *Azospirillum* sp. B510.
(A) EPS production by B510 on NB agar plates containing 0.01% calcofluor white is shown. Control, NB agar plate; $+KNO_3$, NB agar plate containing 10 mM KNO_3; $+NH_4Cl$, NB agar plate containing 10 mM NH_4Cl. B510 was streaked on to these agar plates and incubated for 5 d at 28° C. The photo was taken under UV light. (B) Quantification of biofilm production by crystal violet assay. Incubated B510 in NB media (control), NB media containing 1 mM KNO_3 ($+KNO_3$), NB media containing NH_4Cl ($+NH_4Cl$) in 96 PVC plate wells. Values presented are the average ± SD from four replicates of one plant each. Different letters indicate statistically significant differences between treatments (Student-Newman-Kuels (SNK) test, $p < 0.05$). Similar results were obtained in two independent experiments.

(Naher *et al*., 2018)

Table S1. Bacterial strains and plasmids used in this study

Strain or plasmid	Characteristics	Reference or source
Bacterial strains		
Azospirillum sp. B510	Wild-type strains, Pol^r	Elbertagy et al., 2001
DsRed-tagged *Azospirillum* sp. B510	*Azospirillum* sp. B510 derivative containing pBjGroEL4::DsRed2 planmid insertion, Pol^r, Sm^r	This study
Plasmid		
pBjGroEL4::DsRed2	DsRed transposon delivery vector; Sm^r	Hayashi et al., 2014

Abbreviations used within this table: Pol^r, polymyxin B resistance; Sm^r, streptomycin resistance

Table S2. Measurement of the pH of RG media containing different concentrations of nitrogen sources after 7 d of rice cultivation. Low pH (below 4) is indicated in bold type.

Concentration (mM)	**KNO_3**		**urea**		**NH_4NO_3**		**NH_4Cl**		**NH_4Cl+MES**	
B510	-	+	-	+	-	+	-	+	-	+
0 mM	5.2 (± 0.02)	5.2 (± 0.03)								
0.01 mM	5.2 (± 0.05)	5.2 (± 0.02)	4.7 (± 0.05)	5.3 (± 0.18)	4.9 (± 0.03)	5.2 (± 0.16)	5.6 (± 0.03)	5.5 (± 0.02)	5.6 (± 0.02)	5.6 (± 0.00)
0.1 mM	5.6 (± 0.06)	5.6 (± 0.06)	5.0 (± 0.05)	5.3 (± 0.15)	4.7 (± 0.04)	5.2 (± 0.08)	5.1 (± 0.02)	4.9 (± 0.06)	5.5 (± 0.02)	5.5 (± 0.00)
1 mM	6.3 (± 0.05)	6.3 (± 0.05)	4.5 (± 0.09)	4.8 (± 0.10)	4.2 (± 0.14)	**3.9** (± 0.10)	**3.9** (± 0.13)	**3.9** (± 0.05)	5.5 (± 0.03)	5.5 (± 0.00)
10 mM	6.6 (± 0.03)	6.7 (± 0.02)	7.7 (± 0.11)	8.4 (± 0.17)	**3.0** (± 0.04)	**3.1** (± 0.03)	**3.1** (± 0.16)	**3.4** (± 0.02)	5.5 (± 0.01)	5.5 (± 0.02)

Table S3. Toxicity of NH_4^+ towards *Azospirillum* sp. B510.

NH_4Cl concentration	0 mM	0.01 mM	0.1 mM	1 mM	10 mM
OD_{600}	0.48 ± 0.04	0.69 ± 0.02*	0.63 ± 0.07*	0.67 ± 0.07*	0.68 ± 0.04*

Table S4. The effect of pH on the growth of *Azospirillum* sp. B510 in NB media.

	3	4	5	6	7	8
OD_{600}	0.05 ± 0.03	0.11 ± 0.07	3.37 ± 0.31	3.72 ± 0.09	3.17 ± 0.13	0.23 ± 0.34
